# Efficacy of metformin as an adjuvant therapy in gynecologic malignancies: a meta-analysis of randomized controlled trials

**DOI:** 10.3389/fphar.2026.1752095

**Published:** 2026-03-31

**Authors:** Shasha Zhang, Bin Chen, Xia Hu, Shiquan Hu, Yang Li, Jinbiao Han

**Affiliations:** 1 Department of Gynecology and Obstetrics, West China Second University Hospital, Sichuan University, Chengdu, China; 2 Key Laboratory of Birth Defects and Related Diseases of Women and Children (Sichuan University), Ministry of Education, Chengdu, China; 3 Department of Pediatrics, West China Second University Hospital, Sichuan University, Chengdu, China; 4 Department of Gynecological Nursing, West China Second University Hospital, Sichuan University, Chengdu, China; 5 Department of Gynecological Nursing, Meishan Maternity and Child Healthcare Hospital, Meishan, Sichuan, China; 6 Department of Operating Room Nursing, West China Second University Hospital, Sichuan University, Chengdu, China; 7 Department of Emergency Medicine, Meishan Maternity and Child Healthcare Hospital, Meishan, Sichuan, China

**Keywords:** gynecologic malignancies, metformin, overall survival, progression-free survival, randomized controlled trial

## Abstract

**Introduction:**

Gynecologic malignancies, such as cervical, endometrial, and ovarian cancers, are among the most prevalent and lethal cancers in women worldwide. Preclinical and epidemiological studies suggest that metformin may exert antitumor effects. However, its clinical efficacy in gynecologic malignancies remains uncertain. Hence, exploring the effects of metformin in prolonging progression-free survival (PFS) and overall survival (OS) in gynecological malignancies is crucial for guiding future clinical practice. This systematic review and meta-analysis of randomized controlled trials (RCTs) aimed to assess the effect of metformin combined with standard therapy on PFS and OS in individuals with gynecologic malignancies.

**Methods:**

Embase, the Cochrane Library, Web of Science, and PubMed were searched from database inception to 13 June 2025. RCTs meeting predefined PICOS criteria were included. Two investigators independently screened the studies, extracted data, and assessed the quality of eligible studies. Stata 15.1 was utilized to carry out meta-analyses, and random- or fixed-effects models were selected according to I^2^ values. Subgroup and sensitivity analyses were also performed.

**Results:**

Five RCTs involving 705 patients were included. The overall analyses demonstrated that metformin combined with standard therapy did not significantly improve PFS (hazard ratio [HR] = 0.76, 95% confidence interval [CI]: 0.55–1.03, I^2^ = 36.1%) or OS (HR = 1.20, 95% CI: 0.88–1.62, I^2^ = 0.0%) compared with the control group. Subgroup analyses demonstrated no survival benefits in individuals with cervical or endometrial cancer. Only one study on ovarian cancer suggested that metformin might improve PFS (HR = 0.24, 95% CI: 0.09–0.65). However, the wide CI indicated limited reliability of the results. Sensitivity analyses confirmed the robustness of the findings.

**Conclusion:**

Current evidence from RCTs demonstrates that metformin combined with standard therapy can not significantly improve PFS or OS in individuals with gynecologic malignancies. Further larger, multicenter, long-term RCTs are warranted to evaluate the potential benefits of metformin in individuals with metabolic abnormalities and its combined use with novel therapies.

**Systematic Review Registration:**

https://www.crd.york.ac.uk/PROSPERO/, identifier, CRD2025105994.

## 1 Introduction

Gynecologic malignancies, including ovarian cancer (OC), endometrial cancer (EC), and cervical cancer (CC), are among the most prevalent reproductive system cancers in women worldwide, with high incidence and mortality rates (Sung et al., 2021; Tan et al., 2024; Bray et al., 2024). According to the latest epidemiological data, an estimated 660,000 new cases of CC and 350,000 related deaths were reported globally in 2022. The incidence and mortality rates are 6.8% and 8.1% for CC and 3.4% and 4.8% for OC, respectively. The incidence of EC is approximately 4.3%. Because most individuals with EC present with vaginal bleeding, their prognosis is relatively favorable (Bray et al., 2024). The incidence of CC is increasing in younger individuals, especially those aged 30–59 years. Women aged 30–44 years and 45–59 years account for 36.4% and 46.5% of cases, respectively (Xi et al., 2023). In developed countries in Europe and North America, EC has become the most common gynecologic malignancy. Its incidence is steadily rising, partly due to the prevalence of metabolic syndrome, diabetes mellitus (DM), and obesity (Corr et al., 2025; Lortet-Tieulent et al., 2018; Felix and Brinton, 2018). OC is often diagnosed at advanced stages (stage III–IV) in approximately 80% of patients due to the lack of effective early screening tools. The 5-year survival rates of OC at advanced stages III and IV are 27% and 13%, respectively (Sideris et al., 2024). Despite advances in surgery, radiotherapy, chemotherapy, and molecularly targeted therapies, recurrence and drug resistance remain frequent, leading to limited improvements in prognosis (Konstantinopoulos and Matulonis, 2023; Bogani et al., 2024; Francoeur et al., 2025). Hence, identifying safe, cost-effective, and accessible new adjuvant therapies is imperative.

Metformin is a widely used first-line oral hypoglycemic agent, which is safe and affordable (Bailey and Day, 1989; Ben et al., 2010). Increasing evidence from both preclinical and clinical studies reveals that metformin may exert antitumor effects in individuals with DM (Yao et al., 2019; O'Connor et al., 2024). Epidemiological research has demonstrated that type 2 DM patients receiving metformin have significantly lower risks of cancers and cancer-related mortality compared with non-users (O'Connor et al., 2024; Bowker et al., 2006; Søndergaard et al., 2023). The antitumor mechanisms of metformin are complex, involving both direct and indirect effects. Metformin activates AMP-activated protein kinase (AMPK) and inhibits the PI3K/AKT/mTOR pathway, thereby suppressing the proliferation of tumor cells and promoting apoptosis (Lv and Guo, 2020; Kalender et al., 2010; Saini and Yang, 2018). It also reduces insulin-like growth factor (IGF) levels and blocks tumor growth signals (Birzniece et al., 2022; Nwabo et al., 2022). In addition, metformin can induce Parkin/Pink1-dependent mitophagy and promote apoptosis in CC HeLa cells (Chen J. et al., 2021). It can also downregulate hexokinase 2 via the p53/PDK1/AKT pathway, thereby inhibiting the proliferation of OC cells (Han et al., 2019). Indirectly, metformin mitigates insulin resistance, suppresses hepatic gluconeogenesis, and markedly reduces blood glucose and peripheral insulin levels, thereby restricting energy supply and metabolic demand of tumor cells and creating a less favorable environment for tumor progression (Hua et al., 2020). These findings provide a biological basis for metformin as a potential antitumor agent.

Several clinical studies have assessed the therapeutic effects of metformin in gynecologic malignancies, but their results remain inconsistent. Whether metformin can prolong the survival in patients with gynecological malignancies is still inconclusive. Especially, there is no systematic review to integrate evidence from randomized controlled trials (RCTs). Some observational studies demonstrate that metformin may lower the risk and improve the prognosis of EC and OC among patients with DM (Perez-Lopez et al., 2017; Shi et al., 2019; Kaur et al., 2024). However, RCTs have reported inconsistent findings. For instance, certain studies have reported that metformin may prolong progression-free survival (PFS) in OC (Khalili, 2018), whereas others find no significant benefit (Romero et al., 2025; Zheng et al., 2019). These inconsistent findings may be attributed to limited sample sizes and differences in patient disease types, the dosage and treatment duration of metformin, and therapeutic regimens. Overall, high-quality evidence is still lacking.

Most existing systematic reviews are based on observational studies, which may have a high risk of bias (RoB) and do not sufficiently control for confounders, making it difficult to draw definitive conclusions. In contrast, RCTs, which provide the highest level of clinical evidence, can more directly assess the true efficacy of metformin. To date, no systematic review of RCTs has been conducted in gynecologic malignancies. Hence, the present study systematically searched and appraised the quality of published RCTs following the PRISMA statement. This meta-analysis aimed to examine the effect of metformin combined with standard therapy on PFS and overall survival (OS) in individuals with gynecologic malignancies. The findings may offer evidence-based guidance for its clinical application and inform future research.

## 2 Methods

This study strictly followed the guidelines outlined in the Preferred Reporting Items for Systematic Reviews and Meta-Analyses (PRISMA) statement (Moher et al., 2009). In addition, the study protocol was prospectively registered in the International Prospective Register of Systematic Reviews on 26 May 2025 (registration number CRD 420251059994).

### 2.1 Literature search

Web of Science, Embase, the Cochrane Library, and PubMed were searched from database inception to 13 June 2025, using the search terms: gynecological neoplasms and metformin. The subject headings and free-text terms were combined through Boolean operators to design the search strategies.

### 2.2 Inclusion and exclusion criteria

The inclusion criteria were established based on the PICOS framework (Population, Intervention, Comparison, Outcomes, and Study design): (i) population: individuals with clinically and pathologically confirmed gynecologic malignancies, including CC, OC, or EC; (ii) intervention: standard chemotherapy or chemoradiotherapy, with metformin administered as an adjunctive treatment; (iii) comparator: standard chemotherapy or chemoradiotherapy alone; (iv) outcomes: PFS and OS; (v) study design: RCTs.

Exclusion criteria were as follows: (i) meta-analyses, narrative reviews, or systematic reviews; (ii) editorials, conference abstracts, or case series; (iii) non-English publications; (iv) non-RCTs, such as cohort studies; (v) duplicate publications.

### 2.3 Study selection

All retrieved records were first imported into EndNote 21 for deduplication. Two independent reviewers (ZSS and HX) then screened the titles and abstracts based on the eligibility criteria to exclude clearly irrelevant studies. The full texts of potentially eligible studies were further assessed to determine eligible studies. Any disagreements between the two reviewers were addressed by a third reviewer (HJB). The screening process was presented in a PRISMA flow diagram.

### 2.4 Data extraction

Data were independently extracted by two reviewers (ZSS and HX) using a predesigned data extraction form. Disagreements during the process were resolved by a third reviewer (HJB). Extracted information included three categories: (i) basic study information, such as country or region, year of publication, sample size, study design, and first author; (ii) patient characteristics, including patient age, cancer type (CC, EC, or OC), International Federation of Gynecology and Obstetrics (FIGO) stage, presence of DM, and allocation to intervention or control groups; (iii) outcome measures, mainly including OS and PFS. Hazard ratios (HRs) with 95% confidence intervals (CIs) were collected from each study. If not directly reported, HRs and CIs were estimated from survival curves or relevant statistics. All extracted data were cross-checked by the two reviewers to minimize entry errors and ensure the consistency of the data.

### 2.5 Quality assessment

Two reviewers used the Quality Assessment of Controlled Intervention Studies by National Heart, Lung, and Blood Institute (NHLBI) to assess the risk of bias in the included studies. This tool is a standardized assessment tool specifically designed by NHLBI for RCTs and controlled intervention studies. It covers 14 key items, including random sequence generation, allocation concealment, blinding, baseline comparability, loss to follow-up, and data integrity, and can fully assess the internal authenticity of RCTs. Each item was rated as “Yes,” “No,” or “NA”. Dissents between the two reviewers, if any, were resolved by a third reviewer. The overall quality of the studies was rated as poor (0–5, high RoB), fair (6–10, moderate RoB), and good (11–14, low RoB).

### 2.6 Data analysis

All statistical analyses were conducted using Stata version 15.1. HRs with 95% CIs were used as effect sizes. Fixed-effects models were applied when I^2^ ≤50%, while random-effects models were adopted when I^2^ >50%. Heterogeneity was assessed using the I^2^ statistic. Subgroup and sensitivity analyses were performed to explore potential sources of heterogeneity. The robustness of results was assessed using the leave-one-out method to examine the influence of individual studies on the pooled results. Given that fewer than 10 studies were included, publication bias was not assessed. For studies reporting only survival curves, HRs and 95% CIs were extracted from the curves using previously described methods.

## 3 Results

### 3.1 Search results and study characteristics

**FIGURE 1:**
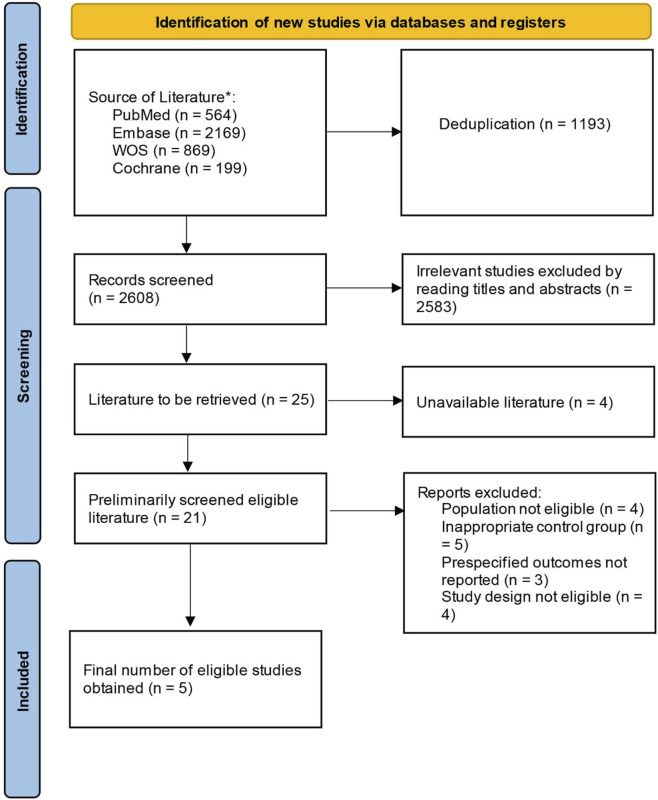
Flow diagram of the literature screening process.

Initially, 3,801 records were identified, including 564 records in PubMed, 2,169 in Embase, 869 in Web of Science, and 199 in Cochrane Library. After excluding duplicates in Endnote, 2,608 records remained. Based on the review of titles and abstracts, 2,583 irrelevant studies were removed, and 25 articles were left for full-text review. Of these, 4 were excluded due to unavailable full texts. Among the remaining 21 studies, 16 were further removed due to ineligible population (n = 4), inappropriate control group (n = 5), no outcomes of interest (n = 3), and ineligible study design (n = 4). Finally, 5 RCTs were included (Khalili, 2018; Romero et al., 2025; Zheng et al., 2019; Bae-Jump et al., 2025; Han et al., 2022) (Figure 1).

### 3.2 Baseline characteristics of included studies

Five RCTs met the inclusion criteria (Khalili, 2018; Romero et al., 2025; Zheng et al., 2019; Bae-Jump et al., 2025; Han et al., 2022). Their main characteristics are summarized in Table 1. Three studies were conducted in North America (two in the United States and one in Canada) and two in Asia (one in Iran and one in China). All studies were published between 2018 and 2025. Three studies focused on OC, one on CC, and one on EC. Patient age ranged from 30 to 80 years, and the disease staging was based on the FIGO classification. There were 705 patients, with 348 receiving standard therapy plus metformin and 356 receiving standard therapy alone. In four trials, the dosage of metformin was 850 mg once or twice daily, while in one trial, the dosage started at 500 mg once daily and was gradually increased to three times daily. Details are presented in Table 1.

**TABLE 1:** Baseline characteristics of the included studies.

First author (surname)	Year of publication	Country/Region	Total sample size	Age (mean ± SD; median [min–max])	Disease name	FIGO stage	Exclusion of diabetes	Name of intervention	Sample size of intervention group	Dose/Frequency	Treatment duration of metformin	Name of control treatment	Sample size of control group	Study design (RCT or cohort)	Outcome variable
Han	2022	Canada	14	MET: 52 (31–74); non_MET: 46 (44–72)	Cervical cancer	Stage IB2–IVA	Yes	Metformin	10	850 mg once daily for 3 days, then 850 mg twice daily during radiotherapy	Starting from the day of random assignment, it continues throughout the entire external radiation therapy period	Chemoradiotherapy	3	RCT	PFS
Bae-Jump	2025	United States	469	NA	Endometrial cancer	Stage III, IVA, IVB, and recurrent endometrial cancer	Not reported	Metformin	234	850 mg twice daily	Starting from the first day of chemotherapy, it continue until disease progression or when further treatment is no longer possible due to intractable events	Chemotherapy + placebo	235	RCT	PFS and OS
Hamedi	2018	Iran	70	MET: 49.7 (36–80); non_MET: 47.5 (30–62)	Ovarian cancer	Epithelial ovarian cancer, Stage I–III	Yes	Metformin	30	500 mg once daily, gradually increased to three times daily	Chemotherapy duration	Chemotherapy	40	RCT	PFS
Romero	2025	United States	108	MET: 64.2 ± 10.6; non_MET: 63.7 ± 10.2	Ovarian cancer	Ovarian cancer, Stage III–IVA (excluding mucinous subtype))	Yes	Metformin	54	850 mg twice daily	During chemotherapy, until 2 years after randomization	Chemotherapy + placebo	54	RCT	PFS and OS
Zheng	2019	China	44	MET: 53.55 ± 9.20; non_MET: 52.88 ± 8.77	Ovarian cancer	Epithelial ovarian cancer, Stage I–IV	Yes	Metformin	20	850 mg once daily	Chemotherapy duration	Chemotherapy	24	RCT	PFS

Met, metformin; non_MET, non-metformin; NA, not available; RCT, randomized controlled trials; PFS, progression-free survival; OS, overall survival.

### 3.3 Risk of bias assessment

Overall, the included studies were of good quality, as most studies had a low risk of bias in randomization, allocation concealment, blinding of participants and investigators, and blinding of outcome assessment. Specifically, the studies by Han (2022), Bae-Jump et al. (2025), Han et al. (2022) demonstrated low overall RoB, with reasonable generation of random sequences, balanced baseline characteristics between groups, appropriate blinding, and adherence to intention-to-treat analysis. In contrast, the studies by Fleming et al. (2025), Hamedi et al. (2018), Khalili (2018), Romero et al. (2025) showed potential bias in certain domains, mainly in allocation concealment, blinding, and incomplete reporting of sample size. The study by Zheng et al. (2019) had moderate RoB due to unclear randomization methods, incomplete reporting of allocation concealment, and lack of blinded outcome assessment.

Among the five included RCTs, two studies (Bae-Jump et al., 2025; Han et al., 2022) explicitly reported the use of intention-to-treat analysis. Attrition rates were below 20% across all studies. Moreover, most trials reported good adherence and similarity of interventions between groups. However, some studies did not sufficiently describe the treatment adherence and had insufficient statistical power for their sample size.

### 3.4 Meta-analysis results

**FIGURE 2:**
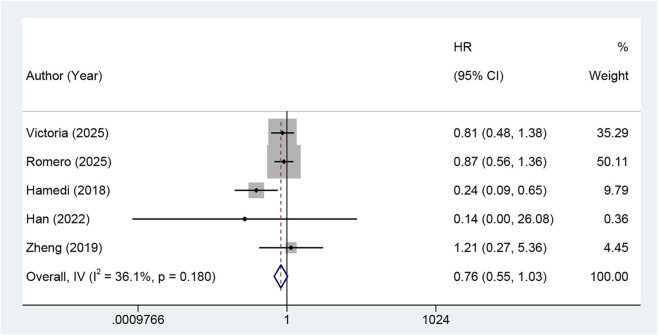
Forest plot of the effect of metformin on progression-free survival in gynecologic cancers (Note: The addition of metformin did not significantly improve PFS. Abbreviations: HR: risk ratio; CI: confidence interval, PFS: progression-free survival).

Five RCTs (Khalili, 2018; Romero et al., 2025; Zheng et al., 2019; Bae-Jump et al., 2025; Han et al., 2022) reported PFS. The pooled results demonstrated no significant difference in PFS between individuals receiving standard therapy combined with metformin and the control group (HR = 0.76, 95% CI: 0.55–1.03, I^2^ = 36.1%, p = 0.180) (Figure 2). Most trials reported negative results. Only the study by Hamedi et al. (2018), OC suggested that metformin might improve PFS (HR = 0.24, 95% CI: 0.09–0.65). However, the wide CI indicated limited reliability of the results. Sensitivity analyses confirmed the robustness of the overall results, and exclusion of any single study did not substantially change the overall estimates.

**FIGURE 3:**
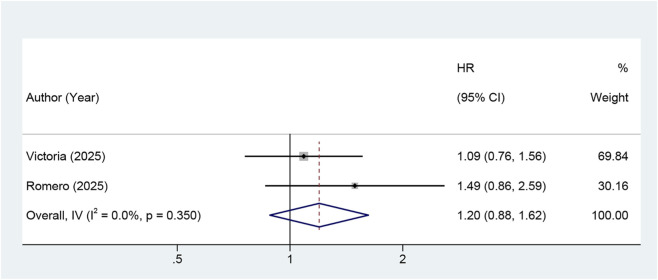
Forest plot of the effect of metformin on overall survival in gynecologic cancers (Note: The addition of metformin did not significantly improve OS. Abbreviations: HR: risk ratio; CI: confidence interval, OS: overall survival).

Two RCTs (Romero et al., 2025; Bae-Jump et al., 2025) reported OS. Neither study revealed the benefits of metformin in improving OS. The pooled analysis indicated no statistically significant difference in OS between individuals receiving standard therapy combined with metformin and the control group (HR = 1.20, 95% CI: 0.88–1.62, I^2^ = 0.0%, p = 0.350) (Figure 3).

**FIGURE 4:**
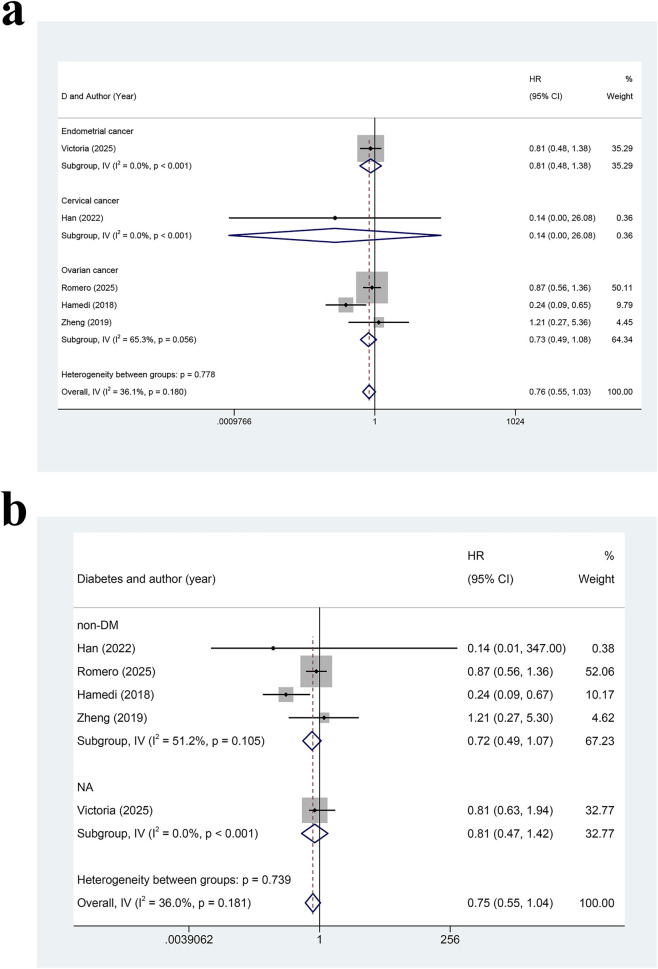
**(a)** Forest plot for subgroup analysis of the effect of metformin on progression-free survival in gynecologic cancers (Note: The addition of metformin did not significantly improve PFS. Abbreviations: HR: risk ratio; CI: confidence interval, PFS: progression-free survival). **(b)** Forest plot for subgroup analysis of the effect of metformin on progression-free survival in patients without diabetes or with unclear diabetic status (Note: The addition of metformin did not significantly improve PFS in patients without DM and with unknown DM status. Abbreviations: HR: risk ratio; CI: confidence interval, PFS: progression-free survival).

Subgroup analyses by cancer type (Figure 4a) demonstrated that three RCTs on OC yielded a pooled HR of 0.73 (95% CI: 0.49–1.08, I^2^ = 65.3%), which was close to statistical significance but remained negative. One RCT on OC (Hamedi et al., 2018) indicated that metformin might improve PFS (HR = 0.24, 95% CI: 0.09–0.65). However, the reliability of the results was limited due to the small sample size. One RCT on EC (Bae-Jump et al., 2025) demonstrated no significant difference (HR = 0.81, 95% CI: 0.48–1.38), and one RCT on CC (Han et al., 2022) also found no significant difference (HR = 0.14, 95% CI: 0.00–26.08). Subgroup analysis by DM status showed no PFS benefit in both individuals without DM and those with unknown DM status (Figure 4b). Notably, one of the five included studies had an intermediate risk. However, sensitivity analysis showed that the exclusion of the study did not change the overall results. These findings indicated that metformin did not improve OS or PFS in the overall population, but it may provide potential benefits in individuals with OC.

## 4 Discussion

This study included five RCTs, involving 705 individuals with gynecological malignancies. The meta-analysis demonstrated that metformin combined with standard therapy did not significantly improve their PFS or OS. In subgroup analyses, no significant survival benefit was found among patients with CC or EC. Only one small-scale RCT on OC suggested that metformin might improve PFS (HR = 0.24, 95% CI: 0.09–0.65), but the robustness of this finding was limited due to the wide CI. Overall, current evidence from RCTs does not support a definitive survival benefit of metformin in individuals with gynecological malignancies.

In contrast, several cohort and retrospective studies have indicated that metformin may exert protective effects against gynecological cancers. For instance, women with DM receiving long-term metformin had lower risks of EC and OC, and the prognosis among metformin users is better (Perez-Lopez et al., 2017; Shi et al., 2019; Kaur et al., 2024). This discrepancy may be attributable to differences in study design. Cohort studies, with larger sample sizes and longer follow-up, can capture indirect antitumor effects of metformin in improving metabolism. However, these studies are more susceptible to confounding factors such as DM control, concomitant medications (such as statins and aspirin), and weight management. These factors may exaggerate its protective effect. In contrast, RCTs usually focus on short-term adjunctive therapy and patients without DM. As a result, they cannot capture the indirect effects of metformin in improving insulin resistance and reducing IGF-1.

Moreover, *in vitro* studies have demonstrated that metformin can activate the AMPK pathway, inhibit PI3K/AKT/mTOR signaling, induce mitochondrial autophagy, and activate p53, thereby directly inhibiting the proliferation of tumor cells (Subbiah et al., 2024; Qiang et al., 2019; Chen Y. H. et al., 2021). However, the dosages used in clinical RCTs (mostly 850 mg once or twice daily) may not reach the effective concentrations found in experimental models. Furthermore, the strong effects of chemotherapy or chemoradiotherapy could mask the marginal benefit of metformin. In addition, tumor cells can undergo adaptive responses through multiple pathways, which may attenuate the direct inhibitory effects of metformin. Consequently, the antitumor activity observed in mechanism studies has not been fully reflected in RCTs.

This study has certain limitations. Firstly, the statistical power was limited as only five RCTs were included, and some had very small sample sizes. This study only included formally published studies, and we did not search clinical trial registry databases such as ClinicalTrials.gov or grey literature (conference abstracts, dissertations, etc.). Accordingly, some high-quality trials that are in progress or have not yet been published may be overlooked. Secondly, most studies were carried out in North America and Asia, with limited data from other regions, thereby limiting the generalizability of the findings observed in this study. Thirdly, among the analyzed cancers, including OC, CC, and EC, there are significant differences in their biological characteristics, the clinical staging criteria, and the dosage and treatment regimen of metformin used in the studies, which may influence the pooled results. Notably, except for one study, all the other included RCTs excluded patients with DM. Therefore, the conclusions of this study are only applicable to individuals without DM. Finally, some studies did not provide sufficient details on treatment adherence, blinding procedures, and sample size, which may also affect the reliability of the results.

In conclusion, this study found that metformin combined with standard therapy could not improve survival outcomes in individuals with gynecological malignancies, despite a potential benefit in OC patients. Given the inconsistency between cohort studies and RCTs, larger-scale, multicenter RCTs with long-term follow-up, particularly targeting individuals with metabolic abnormalities, are needed to assess the effects of long-term administration of metformin. Of the five included studies, four only provided age ranges and medians. Furthermore, this study solely includes RCTs, and our results are different from those reported in previous meta-analyses based on observational studies. This is an important finding of our meta-analysis. Although the statistical power of this study may be low, we believe that providing a comprehensive quantitative meta-analysis offers more accurate effect size estimates than purely descriptive systematic reviews. The findings can provide insights for future research. Therefore, it is impossible to conduct subgroup analyses by age. Future research should explore the effects of metformin combined with immunotherapy or targeted therapy in the treatment of gynecologic malignancies and conduct subgroup analysis by metabolic markers.

## Data Availability

The original contributions presented in the study are included in the article/supplementary material, further inquiries can be directed to the corresponding author.
